# Exogenous oxidized phytosterol may modulate linoleic acid metabolism through upregulation of fatty acid desaturase in rats

**DOI:** 10.1002/lipd.12444

**Published:** 2025-04-09

**Authors:** Tomonari Koyama, Kyoichi Osada

**Affiliations:** ^1^ Department of Agricultural Chemistry School of Agriculture, Meiji University Kawasaki Kanagawa Japan

**Keywords:** cholesterol, fatty acid desaturase, fatty acid metabolism, linoleic acid, oxidized phytosterol, rat

## Abstract

Previous in vitro studies have indicated that oxidized phytosterol (OPS) exhibits some toxicity; however, the harmful effects of OPS on fatty acid metabolism are not completely understood yet. Therefore, this study examined the effects of exogenous phytosterol (PS) and OPS on growth parameters and lipid metabolism in rats. Rats were provided with AIN‐76 basal diet, basal diet +0.5% PS, or basal diet +0.5% OPS. We found that the level of cholesterol and triacylglycerols in the liver was significantly lower in OPS‐fed rats than in basal diet‐fed rats. The ratio of Δ6 desaturation index (20:3(n‐6) + 20:4(n‐6))/18:2(n‐6) in the plasma was significantly higher in the OPS‐fed rats than in the PS‐fed rats. Additionally, the proportion of arachidonic acid (20:4) in the liver was significantly higher in the OPS‐fed group compared with the control group. The mRNA expression levels of Δ6 and Δ5 desaturases were significantly higher in OPS‐fed rats than in basal diet‐fed rats, but remained unchanged in PS‐fed rats. Moreover, the protein level of Δ6 desaturase was significantly higher in both PS‐ and OPS‐fed rats compared with basal diet‐fed rats, while the protein level of Δ5 desaturase tended to be higher only in OPS‐fed rats than in basal diet‐fed rats. Thus, exogenous OPS, but not PS, altered fatty acid composition through the upregulation of mRNA and protein levels of fatty acid desaturation enzymes in the liver. This indicates that exogenous OPS, unlike PS, may modulate the production of eicosanoids from arachidonic acid, potentially promoting allergic reactions, inflammation, and atherosclerosis.

## Graphical abstract

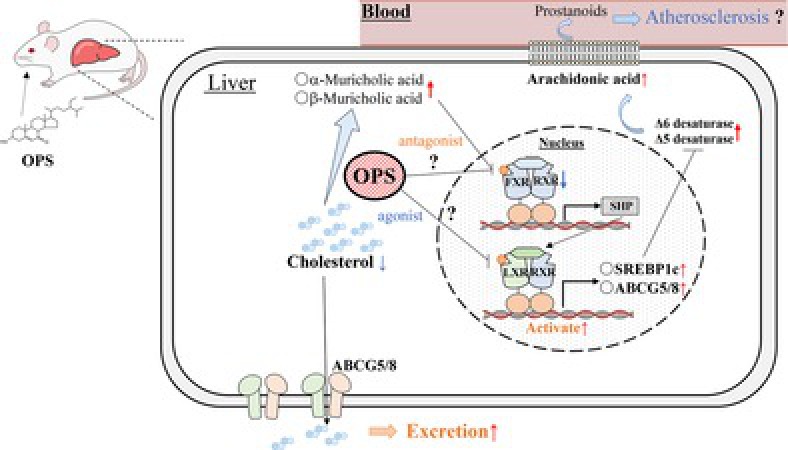

**Abbreviations**
ABCG5: ATP‐binding cassette transporter 5
COX‐2: cyclooxygenase‐2
ELOVL5: fatty acid elongase 5
FID: flame ionization detector
FXR: farnesoid X receptor
HDL: high‐density lipoprotein
HMGCR: HMG‐CoA reductase
LDL: low‐density lipoprotein
LXRα: liver X receptor α
mTOR1: complex 1 of the mammalian target of rapamycin
OPS: oxidized phytosterol
OXC: oxidized cholesterol
PLA_2_
: phospholipase A_2_
PS: phytosterol
RT‐PCR: reverse transcription polymerase chain reaction
SREBP1c: sterol regulatory element binding protein‐1c

## INTRODUCTION

Phytosterols (PS) are abundantly present in plant‐based foods and vegetable oils, and their daily intake level is around 300–500 mg (Hirai et al., 1986; Klingberg et al., 2008) PS are classified into several types based on the differences in their side chains, with the major PS being β‐sitosterol, campesterol, stigmasterol, and brassicasterol. Because PS inhibits the absorption of cholesterol from the intestine by competing with dietary cholesterol for absorption, they can lower blood cholesterol levels and have been reported to reduce the risk of coronary heart disease (Katan et al., 2003; Ostlund, 2004). Because of this hypocholesterolemic function, PS are used in various health food products in many countries.

Cholesterol can get oxidized in common animal‐origin foods due to exposure to oxygen and high temperature and/or ultraviolet irradiation, in processed or stored foods due to reaction with free radicals, and in the body due to reactions with reactive oxygen species and enzymes (Luu et al., 2016). Oxidized cholesterol (OXC) exhibits various harmful effects in the body including cytotoxicity (Sevanian & Peterson, 1986), atherosclerosis (Peng et al., 1985), and carcinogenesis (Morin et al., 1991). In addition, some studies have reported that dietary OXC modulates lipid metabolism, such as inhibition of cholesterol metabolism and promotion of linoleic acid desaturation in rats (Osada et al., 1994, 1995). Similarly, various oxidized phytosterols (OPS) are produced in vegetable oils or plant‐origin foods during heat processing or long‐term storage (Lambelet et al., 2003). The major OPS are the 7α/7β‐hydroxy, 7‐keto, 5,6α/5,6β‐epoxy, and triol forms (Lampi et al., 2002; Oehrl et al., 2001). Because OPS are produced during heating of vegetable oils, high levels of OPS are particularly found in fried foods such as potato chips (Dutta & Appeleqvist, 1997) and French fries (Dutta, 1997). Moreover, OPS level in foods has been reported to increase with microwave heating (Menéndez‐Carreño et al., 2008, Menéndez‐Carreño et al., 2008); thus, consumption of reheated processed foods may lead to higher intake of OPS. A previous study has indicated that OPS comprises 1.9% of the daily PS intake and estimated that the daily intake of OPS ranges from 1.2 to 2.9 mg/day for nonheated foods and 3.5 to 4.2 mg/day for heated foods (Scholz et al., 2015). The study assumed that the maximum dietary exposures of OPS are 13 and 130 mg/day at oxidation rates of 0.1% and 1%, respectively (Scholz et al., 2015).

Grandgirard et al. administered rats with OPS intragastrically and found that it was absorbed into the lymph from the intestine (Grandgirard et al., 1999). Additionally, mice administered OPS at 0.2 g/kg were found to have OPS in the liver, plasma, and brain (Wang et al., 2021). Tomoyori et al. reported that oxidized campesterol accounts for ~15.9% of absorbed OPS in mice, whereas oxidized β‐sitosterol accounts for ~9.1%; meanwhile, campesterol and β‐sitosterol comprise ~5.5% and 2.2% of absorbed PS, respectively (Tomoyori et al., 2004). This indicates that the absorptivity of OPS from the intestine is higher than that of PS. OPS, like OXC, has exhibited specific deleterious biological activities including cytotoxicity (Gao et al., 2015; Ryan et al., 2005) and inflammation in many in vitro studies (Alemany et al., 2013). Moreover, OPS has been shown to possess pro‐atherogenic effects (Plat et al., 2014) and inhibit cholesterol metabolism in in vivo studies (Liang et al., 2011). However, most of the effects of OPS have been demonstrated in in vitro studies, and its effects in in vivo studies require further elucidation.

Because OPS is structurally similar to OXC, it may also influence lipid metabolism. Previously, OPS has been reported to affect cholesterol metabolism by downregulating the expression of HMG‐CoA reductase (HMGCR) and SREBP2 genes involved in cholesterol synthesis and the ABCG5 gene of sterol excretion in vivo (Liang et al., 2011) and in vitro studies (Laparra et al., 2015). However, the effects of OPS on fatty acid metabolism remain unclear. Therefore, in this study, we aimed to compare the effects of exogenous PS and OPS on growth parameters, lipid metabolic parameters, and desaturation of essential fatty acids using rats as an animal model.

## MATERIALS AND METHODS

### Preparation and analysis of OPS

PS (Tama Biochemistry Co., Ltd., Tokyo, Japan) contained 4.7% brassicasterol, 23.5% stigmasterol, 25.9% campesterol, and 40.0% β‐sitosterol. PS was heated in an electric oven at 180°C for 8 h and then dissolved in diethyl ether, transferred to a silica gel column (Silica gel 60; Nacalai Tesque, Inc., Kyoto, Japan; 50 × 650 mm), and fractionated by successive elution of diethyl ether and methanol. The fraction eluted by methanol was dried using a rotary evaporator, and the resultant powder was used in the animal experiment as purified OPS.

PS and OPS composition was analyzed using Agilent 5975C series GC/MSD (Agilent Technologies, Inc., Santa Clara, CA, USA) with Zebron ZB‐1 plus (0.25 mm × 60 m, 0.25 μm; Phenomenex, Ltd., Torrance, CA, USA). The conditions were set as follows: The flow rate of helium was 1.5 mL/min. Samples were injected at an oven temperature of 270°C. Ion source temperature was 230°C, while quadrupole temperature was 150°C. The mass spectra were measured within a mass range of 100–600 m/z. Ionization energy was 70 eV. The mass spectra of OPS were decided following the studies of Menéndez‐Carreño, Ansorena, and Astiasarán (2008), Menéndez‐Carreño, García‐Herreros, et al. (2008), Conchillo et al. (2005), and Leal‐Castañeda et al. (2015).

### Animals and diet

The animal experiments were conducted according to the guidelines of the Ethical Committee of Experimental Animal Care at Meiji University (approval code: MUIACUC2020‐01).

Male Wistar rats (3‐week‐old; JAPAN SLC, Inc., Shizuoka, Japan) were housed individually in a temperature‐(22–24°C) and light‐controlled (0:00–5:00 and 17:00–24:00) room. After acclimatization for a week, 21 rats were equally divided into three groups: group C was fed the AIN‐76 basal diet, group P was fed the basal diet supplemented with 0.5% PS, and group O was fed the basal diet supplemented with 0.5% OPS. In the previous study (Koyama et al., 2024), male Wistar rats (4 weeks old) were fed a diet containing 0.25% PS or OPS for 4 weeks. Therefore, we fed the rats relatively high levels of PS or OPS for a short period to clarify the exact effects of exogenous PS or OPS on lipid metabolism, using male Wistar rats (4 weeks old) as in the previous study (Koyama et al., 2024), while taking into account the effects on lipid homeostasis.

The diets were prepared according to the formula recommended by the American Institute of Nutrition (American Institute of Nutrition, 1977), and their detailed compositions are shown in Table 1. The rats were pair‐fed by adjusting their daily food consumption to match the intake of group O. After 7 days, the rats were anesthetized using isoflurane and bled from the heart; various tissues were then quickly excised. Plasma was obtained by centrifuging blood and allowed to clot at room temperature. These samples were kept at −80°C until further analysis. For RNA analysis, part of the liver sample was immersed in RNAlater solution at 4°C for 1 day before cold storage.

**TABLE 1:** Diet composition.

Component	Group		
	C (%)	P (%)	O (%)
Corn starch^a^	15	15	15
Casein^b^	20	20	20
Sucrose^c^	48.425	47.925	47.925
High linoleic safflower oil^d^	5	5	5
Cellulose^e^	5	5	5
Mineral mix. (AIN76)^f^	5	5	5
Vitamin mix. (AIN76)^g^	1	1	1
Choline bitarttrate^h^	0.2	0.2	0.2
DL‐Methionine^i^	0.3	0.3	0.3
Phytosterol^j^	0	0.5	0
Oxidized phytosterol	0	0	0.5
Cholic acid sodium salt^k^	0.075	0.075	0.075

Abbreviations: C, rats fed control diet; P, rats fed phytosterol; O, rats fed oxidized phytosterol.

^a^ Nihon Shokuhin Kako Co., Ltd.

^b^ Feed One Co., Ltd.

^c^ Mitsui. DM Sugar Co., Ltd.

^d^ Nisshin OilliO Group, Ltd.

^e^ Feed One Co., Ltd.

^f^ Oriental Yeast Co., Ltd.

^g^ Oriental Yeast Co., Ltd.

^h^ Nacalai Tesque, Inc.

^i^ Nacakai Tesque, Inc.

^j^ Tama Biochemistry Co., Ltd.

^k^ Nacalai Tesque, Inc.

### Analyses of plasma and liver lipid levels

The levels of plasma triacylglycerols, total cholesterol, and high‐density lipoprotein (HDL) cholesterol were measured using commercial kits (Fujifilm Wako Pure Chemical Corporation, Osaka, Japan). Liver lipids were extracted by the method described by Folch et al. (1951), while liver triacylglycerols and cholesterol were quantified according to the methods described by Ide et al. (1978) and the method of Liebermann‐Burchard reaction (Sugano et al., 1988), respectively.

### Analyses of fatty acid composition of plasma and liver lipids

The fatty acid composition of plasma and liver lipids was analyzed as methyl ester derivations after saponification using the GC‐14B gas chromatograph (Shimadzu Co., Kyoto, Japan) equipped with a flame ionization detector (FID) and TC WAX column (0.25 mm × 30 m, 0.25 μm; GL Sciences, Tokyo, Japan). The GC conditions were set as follows: oven temperature, 210°C (Isothermal temperature condition); injector temperature, 250°C; and flow rate of nitrogen, 1.0 mL/min.

### Analyses of fecal lipids

Fecal neutral steroids were analyzed by gas chromatograph GC‐2025 (Shimadzu Co., Kyoto, Japan) equipped with an FID and ZB‐5MS (0.25 mm × 60 m, 0.25 μm, Phenomenex, CA, USA), following the methods of Sugano et al. (1988); 5α‐cholestane was used as the internal standard. The GC conditions were set as follows: oven temperature, 270°C (Isothermal temperature condition); injector temperature, 300°C; and flow rate of nitrogen, 1.0 mL/min.

Acidic steroids were analyzed by GC‐14B (Shimadzu Co., Kyoto, Japan) with FID and TC‐5 column (0.25 mm × 30 m, 0.25 μm, GL Sciences, Tokyo, Japan) and using 23‐nor‐deoxycholic acid as the internal standard, following the methods of Sugano et al. (1988). The GC conditions were set as follows: oven temperature, 230–270°C (2°C/min temperature elevation); injector temperature, 310°C; and flow rate of nitrogen, 1.5 mL/min.

### RNA extraction

Total RNA was extracted from rat liver tissue using Sepasol‐RNA I Super G (Nacalai Tesque, Inc., Kyoto, Japan). RNA concentration was determined by measuring the absorbance at 260 nm using a Nanodrop Lite spectrophotometer (Thermo Fisher Scientific, Waltham, MA, USA).

### Oligonucleotide primer sequences

The primers for reverse transcription polymerase chain reaction (RT‐PCR) of rat Δ5 and Δ6 desaturase genes were designed using the Primer3Plus software (http://www.bioinfor‐ matics.nl/cgi‐bin/primer3plus.cgi). The primers, synthesized by Eurofins Genomics K.K. (Tokyo, Japan), were designed to flank known or putative introns of these genes, thereby preventing the amplification of any contaminating genomic DNA. The primer sequences were as follows: Δ5 desaturase (Gene ID: 84575), forward 5′‐GATGAACCATATCCCCATGC‐3′ and reverse 5′‐CTTGGCGCACAGAGATTGTA‐3′; Δ6 desaturase (Gene ID: 83512), forward 5′‐ATCTGCCCTACAACCACCAG‐3′ and reverse 5′‐GTGTGACCCACACAAACCAG‐3′; fatty acid elongase 5 (ELOVL5, Gene ID: 171400), forward 5′‐TCTTCTGTCAGGGAACAGGC‐3′ and reverse 5′‐CGAGAGGCCCCTTTCTTGTT‐3′; sterol regulatory element binding protein‐1c (SREBP‐1c, Gene ID: 78968), forward 5′‐GGAGCCATGGATTGCACATT‐3′ and reverse 5′‐AGGAAGGCTTCCAGAGAGGA‐3′; ATP‐binding cassette transporter 5 (ABCG5, Gene ID: 114628), forward 5′‐ATGGCCTGTACCAGAAGTGG‐3′ and reverse 5′‐AGCGGCAGAGAAGTATCCAA‐3′; ABCG8 (Gene ID: 155192), forward 5′‐CGAGCCTCTGAGCCTGTAAC‐3′ and reverse 5′‐GGGCTGGATAGGATCTGACA‐3′; liver X receptor α (LXRα, Gene ID: 58852), forward 5′‐AGACATCGCGGAGGTACAAC‐3′ and reverse 5′‐GGCTCACCAGCTTCATTAG‐3′; farnesoid X receptor (FXR, Gene ID: 60351), forward 5′‐TGGAGTGGTGTTCGAAATGA‐3′ and reverse 5′‐AGGAGGAGATCTGTGGCTGA‐3′; cyclooxygenase‐2 (COX‐2, Gene ID: 29527), forward 5′‐TACCCGGACTGGATTCTACG‐3′ and reverse 5′‐AAGTTGGTGGGCTGTCAATC‐3′; phospholipase A_2_ (PLA_2_, Gene ID: 360426) forward 5′‐ATGACTTCCCTGGGTTTGGTGATGGAG‐3′ and reverse 5′‐ACAGATGGTGGGCCACTTCCCAGA‐3′; β‐actin (Gene ID: 81822), forward 5′‐AGCCATGTACGTAGCCATCC‐3′ and reverse 5′‐CTCTCAGCTGTGGTGGTGAA‐3′. β‐actin was used as a control.

### Real‐time quantitative polymerase chain reaction

RNA (1 μg) was incubated at 65°C for 5 min and then quickly cooled on ice. Reverse transcription of RNA was performed using a ReverTra Ace qPCR RT Master Mix (Toyobo Co., Ltd., Osaka, Japan) and by heating the sample to 37°C for 15 min, followed by reheating at 98°C for 5 min. An aliquot of the generated cDNA samples was mixed with 4.5 μL of THUNDERBIRD Next SYBR qPCR MIX (Toyobo Co., Ltd., Osaka, Japan) in the presence of 0.3 μmol of each of the forward and reverse primers for β‐actin and the target gene. This reaction mix was then subjected to the following cycling conditions in a PIKOREAL 96 Real‐Time PCR System (Thermo Fisher Scientific, Waltham, MA, USA): 1 cycle at 95°C for 1 min, followed by 40 cycles each of 95°C for 15 s, 58.4°C for 15 s, and 72°C for 30 s. The results (fold changes) were expressed as relative folds by comparing the amount of RNA of the target gene to that of β‐actin as an internal control, as determined by the equation 2C^(t)target+C(t)GAPDH^.

### Western blot analysis

The protein levels of enzymes involved in fatty acid desaturation in the liver were analyzed by western blotting. Liver tissue lysates were prepared by lysis with the RIPA buffer (Nacalai Tesque, Inc., Kyoto, Japan). Protein concentrations were measured using Protein Assay CBB Solution (Nacalai Tesque, Inc., Kyoto, Japan). Protein extracts were separated using 12% SDS‐PAGE and transferred to a polyvinylidene difluoride membrane (0.45 μm; GVS North America, Inc., Sanford, ME, USA). The membrane was then blocked in Ez Block Chemi (ATTO Corporation, Tokyo, Japan) for 1 h and then incubated with rabbit anti‐Δ5 desaturase (Cloud‐Clone Corp., TX, USA; 1:5000 vol/vol dilution), mouse anti‐Δ6 desaturase (Proteintech Group, Inc., IL, USA; 1:5000 vol/vol dilution), mouse anti‐ELOVL5 (Santa Cruz Biotechnology, Inc., TX, USA; 1:5000 vol/vol dilution), rabbit anti‐COX‐2 (WUHAN HUAMEI BIOTECH Co., Ltd.; Bukan, China; 1:1000 vol/vol dilution), or mouse anti‐β‐actin (Proteintech Group, Inc., IL, USA; 1:5000 vol/vol dilution) antibody as primary antibody for 24 h at 4°C. The membranes were subsequently washed with the washing solution for 30 min and then incubated with a secondary antibody (Δ6 desaturase, ELOVL5, and β‐actin: goat anti mouse IgG‐HRP, 1:10000 vol/vol dilution; Δ5 desaturase: goat anti rabbit IgG‐HRP, 1:20000 vol/vol dilution; COX‐2: mouse anti rabbit IgG‐HRP, 1:10000 vol/vol dilution) for 1 h at 25°C.

After washing with the washing solution, the signal was detected using the ChemiDoc‐It 410 Imaging System (Ultra Violet Products, Ltd., Cambridge, UK) after incubating with ImmunoStar LD (Fujifilm Wako Pure Chemical Corporation, Osaka, Japan) as the luminescent reagent. The signal of each enzyme was analyzed using CS Analyzer (ATTO Corporation, Tokyo, Japan), and the intensity of each signal was standardized to the signal of β‐actin. The fold increase or fold decrease for each protein was determined as compared to the relevant intensity in group C (set equal to 1).

### Effect of PS or OPS on micellar solubility of cholesterol in vitro

A mixed micellar solution containing 6.6 mM sodium taurocholate (Nacalai Tesque, Inc., Kyoto, Japan), 0.6 mM phosphatidylcholine (from Egg Yolk, Nacalai Tesque, Inc., Kyoto, Japan), 132 mM NaCl, 40 mg trilinolenin (Olbracht Serdary Research Laboratories, Toronto, Canada), PS or OPS (1 or 5 mg), 16 mg cholesterol (Nacalai Tesque, Inc., Kyoto, Japan), and 15 mM sodium phosphate at pH 7.4 was prepared by sonication according to the method described by Ogino et al. (2007). The prepared micellar solution was incubated for 1 h at 37°C and then centrifuged at 1000 g for 10 min. The supernatant was filtered using a 220‐nm filter (Membrane Solutions LLC, TX, USA). Lipids were extracted from the filtered solutions following the method described by Folch et al. (1951). Thereafter, the levels of α‐linolenic acid and steroids were analyzed by GC according to the methods described in Sections “Animals and diet” and “Analyses of fecal lipids.”

### Statistical analysis

All data are expressed as the mean ± standard error (SE). Statistical analyses for the animal experiment results were conducted using one‐way analysis of variance and the Tukey–Kramer test to evaluate significant differences among the means of the three groups. The results of the micellar solubility of cholesterol were analyzed using one‐way analysis of variance and the Dunnett test to evaluate significant differences from group C. Differences were considered significant at *p* < 0.05.

## RESULTS

### Composition of prepared OPS

The prepared OPS contained various oxidized derivatives of stigmasterol, campesterol, and β‐sitosterol. Among these, 7‐hydroxy derivatives were present in the highest amount, followed by 5,6‐epoxy, 7‐keto, and triol derivatives. The level of oxidized brassicasterol was very low. The detailed composition of the prepared OPS is shown in Table 2.

**TABLE 2:** Composition of oxidized phytosterols.

Component	(%)
**Oxidized campesterol**	
7α‐hydroxy campesterol	4.9
7β‐hydroxy campesterol	9.3
5,6β‐epoxy campesterol	5.3
5,6α‐epoxy campesterol	2.1
Campestanetriol	2.0
7‐keto campesterol	2.2
**Oxidized stigmasterol**	
7α‐hydroxy stigmasterol	6.6
7β‐hydroxy stigmasterol	8.2
5,6β‐epoxy stigmasterol	2.2
5,6α‐epoxy stigmasterol	3.2
Stigmastanetriol	1.2
7‐keto stigmasterol	2.1
**Oxidized β‐sitosterol**	
7α‐hydroxy β‐sitosterol	10.2
7β‐hydroxy β‐sitosterol	10.9
5,6β‐epoxy β‐sitosterol	3.7
5,6α‐epoxy β‐sitosterol	3.4
β‐sitostanetriol	1.9
7‐keto β‐sitosterol	3.1
**Oxidized brassicasterol**	
7α‐hydroxy brassicasterol	0.3
Unknown	19.3

### Effect of exogenous PS or OPS on growth parameters

No significant differences were observed in final body weight, weight gain, and liver weight between the three groups (Table 3). The weights of epididymal and mesenteric white adipose tissues were also not significantly different between the three groups. However, the weight of perirenal white adipose tissue was significantly lower in group O than in group P. Consequently, the total weight of white adipose tissue was significantly lower in group O compared with groups C and P.

**TABLE 3:** Effect of dietary phytosterol or oxidized phytosterol on growth parameters.

	Group		
	C	P	O
Food intake (g/day)	16.6 ± 0.3	16.4 ± 0.3	15.9 ± 0.2
Initial body weight (g)	121 ± 2	121 ± 2	121 ± 2
Final body weight (g)	144 ± 2	142 ± 2	141 ± 2
Weight gain (g)	23.7 ± 0.8^a^	22.7 ± 0.9^ab^	20.9 ± 1.0^b^
Liver weight (g/100 g body weight)	4.32 ± 0.2	4.25 ± 0.06	4.44 ± 0.04
WAT weight			
Epididymal WAT (g/100 g body weight)	0.96 ± 0.03	0.96 ± 0.04	0.81 ± 0.05
Perirenal WAT (g/100 g body weight)	0.60 ± 0.03^ab^	0.63 ± 0.04^a^	0.49 ± 0.03^b^
Mesenteric WAT (g/100 g body weight)	0.97 ± 0.10	0.96 ± 0.05	0.88 ± 0.08
Total WAT (g/100 g body weight)	2.53 ± 0.10^a^	2.54 ± 0.06^a^	2.18 ± 0.08^b^

*Note*: Data are presented as the mean ± SE of seven rats in each group. ^ab^Values without a common superscript letter are significantly different at *p* < 0.05. Abbreviations are the same as those in Table 1.

Abbreviation: WAT, white adipose tissue.

### Effect of exogenous PS or OPS on plasma and liver lipid levels

Plasma triacylglycerol levels were not significantly different between the three groups (Table 4). However, plasma total cholesterol level was significantly lower in group O compared with groups C and P. Furthermore, plasma HDL cholesterol level was significantly lower in group O than in group P.

**TABLE 4:** Effect of dietary phytosterol or oxidized phytosterol on plasma and liver lipid levels.

	Group		
	C	P	O
**Plasma**		(mg/dL)	
Triacylglycerol	33.9 ± 5.3	33.3 ± 5.0	23.2 ± 2.8
Total cholesterol	147 ± 6^a^	143 ± 4^a^	126 ± 5^b^
HDL cholesterol	87.5 ± 7.7^ab^	93.4 ± 3.3^a^	71.4 ± 4.0^b^
**Liver**		(mg/g)	
Triacylglycerol	7.99 ± 0.34^a^	7.68 ± 0.43^a^	5.42 ± 0.26^b^
Total cholesterol	4.29 ± 0.06^a^	3.59 ± 0.22^b^	3.17 ± 0.04^b^

*Note*: Data are presented as the mean ± SE of seven rats in each group. ^ab^Values without a common superscript letter are significantly different at *p* < 0.05. Abbreviations are the same as those in Table 1.

Group O also exhibited a significantly lower liver triacylglycerol level compared with groups C and P. Additionally, liver total cholesterol levels were significantly lower in groups P and O than in group C.

### Effects of exogenous PS or OPS on fatty acid composition in the plasma

Compared with group P, group O showed a significantly higher proportion of docosapentaenoic acid (22:5) (Table 5). The ratio of Δ6 desaturation index (20:3(n‐6) + 20:4(n‐6))/18:2(n‐6) was also significantly higher in group O than in group P.

**TABLE 5:** Effect of dietary phytosterol or oxidized phytosterol on plasma fatty acid composition.

		Group		
Fatty acid		C	P	O
			(%)	
14:0		1.94 ± 0.38	1.85 ± 0.34	1.85 ± 0.23
16:0		19.5 ± 1.0	19.2 ± 0.9	18.4 ± 1.04
16:1	(n‐7)	3.40 ± 0.42	3.77 ± 0.41	3.27 ± 0.21
18:0		8.99 ± 0.36	7.74 ± 0.33	8.67 ± 0.58
18:1	(n‐9)	8.57 ± 0.42	8.71 ± 0.26	7.98 ± 0.82
18:2	(n‐6)	11.3 ± 0.4	11.4 ± 1.1	10.4 ± 0.5
18:3	(n‐6)	0.61 ± 0.06	0.63 ± 0.06	0.71 ± 0.09
20:3	(n‐6)	0.30 ± 0.02	0.28 ± 0.04	0.27 ± 0.02
20:4	(n‐6)	21.5 ± 1.1	19.6 ± 1.8	22.0 ± 1.5
22:4	(n‐6)	0.93 ± 0.15	0.64 ± 0.11	0.99 ± 0.23
22:5	(n‐3)	35.6 ± 1.4^ab^	32.5 ± 2.8^a^	34.5 ± 2.0^b^
22:6	(n‐3)	1.39 ± 0.19	1.03 ± 0.18	1.98 ± 0.25
			Ratio	
**Δ6D index**	(20:3 + 20:4)/18:2	1.96 ± 0.08^ab^	1.78 ± 0.13^a^	2.29 ± 0.03^b^
**Δ5D index**	(20:4)/(20:3)	71.3 ± 3.7	79.3 ± 8.9	71.3 ± 10.7

*Note*: Data are presented as the mean ± SE of seven rats in each group. ^ab^Values without a common superscript letters are significantly different at *p* < 0.05. Abbreviations are the same as those in Tables 1 and 5.

### Effects of exogenous PS or OPS on fatty acid composition in the liver and liver

The proportion of palmitoleic acid (16:1) was significantly lower in group P compared with group C, and it tended to be lower in group O compared with P (Table 6, *p* = 0.098). The proportion of oleic acid (18:1) tended to be higher in group O than in group C (*p* = 0.092). The proportion of arachidonic acid (20:4), on the other hand, was significantly higher in group O than in group C. Moreover, the ratio of Δ6 desaturation index and Δ5 desaturation index (20:4(n‐6)/20:3(n‐6)) tended to be higher in group O than in group P (*p* = 0.085 and *p* = 0.056, respectively).

**TABLE 6:** Effect of dietary phytosterol or oxidized phytosterol on liver fatty acid composition.

		Group		
Fatty acid		C	P	O
			(%)	
14:0		0.34 ± 0.03	0.36 ± 0.06	0.22 ± 0.03
16:0		0.15 ± 0.01	0.16 ± 0.02	0.16 ± 0.02
16:1	(n‐7)	20.1 ± 0.9^a^	15.7 ± 1.3^b^	17.0 ± 0.55^a^
18:0		15.8 ± 1.5	17.2 ± 0.6	19.1 ± 1.0
18:1	(n‐9)	13.2 ± 1.1	10.3 ± 0.9	9.62 ± 1.16
18:2	(n‐6)	14.5 ± 1.3	13.2 ± 1.1	12.8 ± 1.2
18:3	(n‐6)	0.18 ± 0.01	0.22 ± 0.05	0.15 ± 0.02
20:3	(n‐6)	0.34 ± 0.01	0.40 ± 0.05	0.39 ± 0.03
20:4	(n‐6)	20.6 ± 0.7^a^	22.2 ± 0.5^ab^	24.4 ± 0.7^b^
22:4	(n‐6)	0.93 ± 0.04	0.99 ± 0.11	1.04 ± 0.07
22:5	(n‐3)	1.86 ± 0.13	1.64 ± 0.14	1.97 ± 0.06
22:6	(n‐3)	6.49 ± 0.50	6.58 ± 0.55	7.05 ± 033
			Ratio	
**Δ6D Index**	(20:3 + 20:4)/18:2	1.58 ± 0.14	1.48 ± 0.13	2.04 ± 0.21
**Δ5D Index**	(20:4)/(20:3)	63.6 ± 2.6	54.5 ± 2.7	69.1 ± 5.3

*Note*: Data are presented as the mean ± SE of seven rats in each group. ^ab^Values without a common superscript letter are significantly different at *p* < 0.05. Other abbreviations are the same as those in Table 1.

Abbreviations: Δ5D, delta 5 desaturation index; Δ6D, delta 6 desaturation index.

### Effect of exogenous PS or OPS on the levels of neutral and acidic steroids in feces

The level of coprostanol was not significantly different between the three groups (Table 7). However, the levels of cholesterol and total neutral steroids were significantly higher in group O than in groups C and P.

**TABLE 7:** Effect of dietary phytosterol or oxidized phytosterol on fecal neutral and acidic steroid levels.

	Group		
	C	P	O
Dried fecal weight (g/day)	2.34 ± 0.05	2.16 ± 0.16	2.15 ± 0.27
Neutral steroids (mg/day)			
Coprostanol	1.22 ± 0.16	1.13 ± 0.24	0.60 ± 0.12
Cholesterol	2.18 ± 0.19^a^	3.65 ± 0.38^a^	8.60 ± 0.89^b^
Total neutral steroids	3.61 ± 0.13^a^	4.78 ± 0.43^a^	9.44 ± 0.70^b^
Acidic steroids (mg/day)			
Cholic acid	2.42 ± 0.15	2.35 ± 0.22	2.88 ± 0.41
α‐mulicholic acid	0.91 ± 0.07	1.53 ± 0.21	1.62 ± 0.23
β‐mulicholic acid	1.85 ± 0.15^a^	1.78 ± 0.27^a^	3.13 ± 0.31^b^
Lithocholic acid	0.47 ± 0.06^a^	0.30 ± 0.04^a^	0.82 ± 0.12^b^
Deoxycholic acid	1.48 ± 0.19^a^	0.72 ± 0.12^b^	1.16 ± 0.08^ab^
Hyodeoxycholic acid	1.43 ± 0.07	1.34 ± 0.24	1.92 ± 0.25
Ursodeoxycholic acid	0.96 ± 0.12	1.00 ± 0.20	2.00 ± 0.37
Total acidic steroids	9.20 ± 0.42	8.89 ± 1.08	12.9 ± 1.51

*Note*: Data are presented as the mean ± SE of seven rats in each group. ^ab^Values without a common superscript letters are significantly different at *p* < 0.05. Abbreviations are the same those as in Table 1.

The levels of fecal cholic acid and hyodeoxycholic acid were not significantly different between the three groups. However, the levels of β‐muricholic acid and lithocholic acid were significantly higher in group O than in groups C and P. Group O showed a higher level of α‐muricholic acid than group C (*p* = 0.064). The ursodeoxycholic acid level was significantly higher in group O compared with group C and tended to be lower in group O compared with group P (*p* = 0.054). The level of deoxycholic acid was significantly lower in group P than in group C. Moreover, the level of total acidic steroids tended to be lower in group O than in group P (*p* = 0.077).

### Effect of exogenous PS or OPS on the mRNA expressions and protein levels of enzymes involved in linoleic acid metabolism in the liver

**FIGURE 1:**
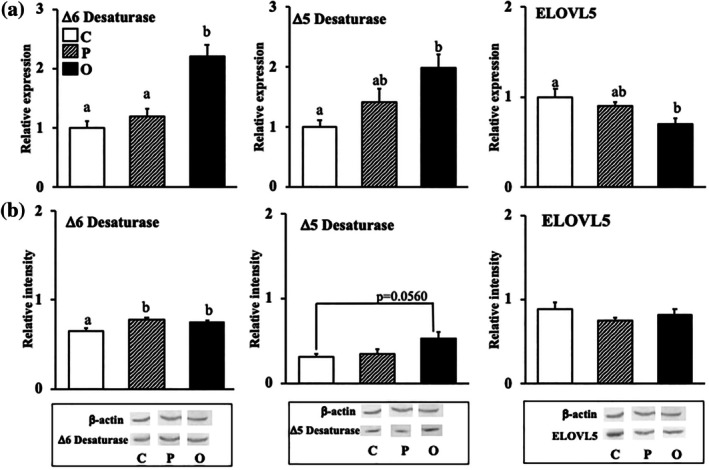
Effect of exogenous phytosterol or oxidized phytosterol on the mRNA expression and protein levels of enzymes involved in linoleic acid metabolism in the liver. (a) mRNA expression level; (b) Protein level. Data are presented as the mean ± SE of seven rats in each group. ^ab^Values without a common superscript letter are significantly different at *p* < 0.05. Abbreviations are the same as those in Table 1.

The mRNA expression level of Δ6 desaturase was significantly higher in group O than in groups C and P (Figure 1). Moreover, the mRNA expression level of Δ5 desaturase was significantly higher in group O compared with group C. However, the mRNA expression level of ELOVL5 was significantly lower in group O than in groups C and P.

The protein level of Δ6 desaturase was significantly higher in groups P and O than in group C. Meanwhile, the protein level of Δ5 desaturase tended to be higher in group O than in group C (*p* = 0.056). The ELOVL5 protein level showed no significant difference between the three groups.

### Effect of exogenous PS or OPS on the mRNA expressions and protein levels of enzymes involved in arachidonic acid metabolism in the liver

**FIGURE 2:**
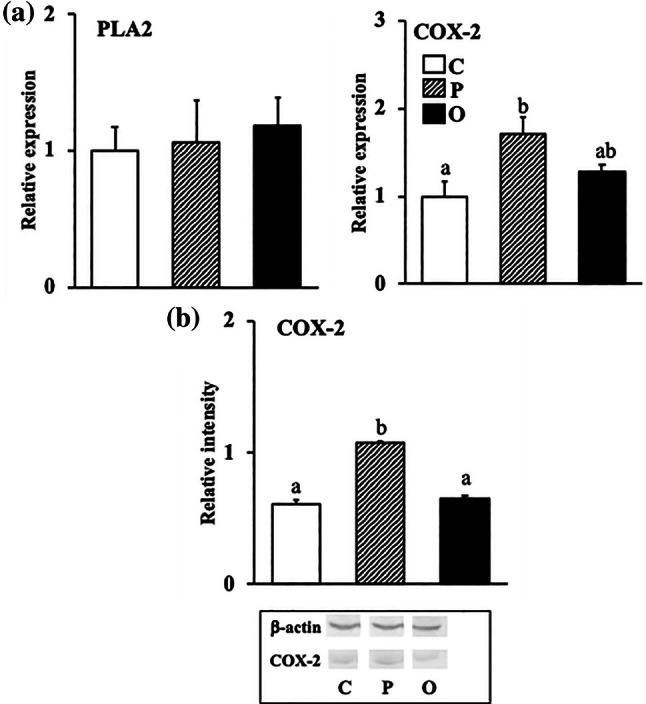
Effect of exogenous phytosterol or oxidized phytosterol on mRNA expression and protein levels of enzymes involved in arachidonic acid metabolism in liver. (a) mRNA expression level; (b) Protein level. Data are presented as the mean ± SE of seven rats in each group. ^ab^Values without a common superscript letter are significantly different at *p* < 0.05. Abbreviations are the same as those in Table 1.

The mRNA expression level of PLA_2_ was not significantly different between the three groups (Figure 2), while the mRNA expression level of COX‐2 was significantly higher in group P compared with group C, but not significantly different from that in group O.

Meanwhile, the protein level of COX‐2 in group P was significantly higher than that in group C but not significantly different from that in group O.

### Effect of exogenous PS or OPS on the mRNA expression of ATP‐binding cassette transporters involved in the excretion of cholesterol in the liver

**FIGURE 3:**
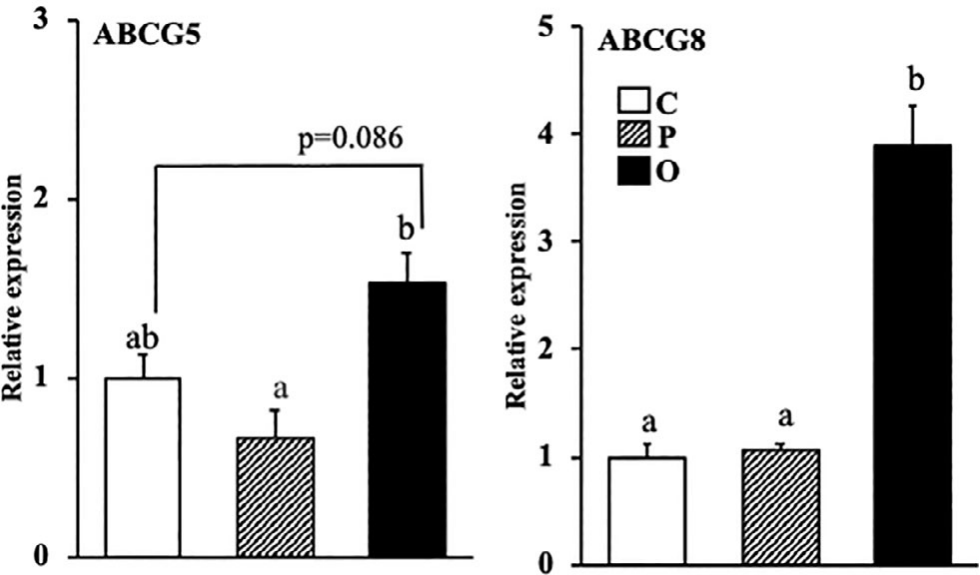
Effect of exogenous phytosterol or oxidized phytosterol on mRNA expression of ATP‐binding cassette transporters involved in the excretion of cholesterol in the liver. Data are presented as the mean ± SE of seven rats in each group. ^ab^Values without a common superscript letter are significantly different at *p* < 0.05. Abbreviations are the same as those in Table 1.

The mRNA expression level of ABCG5 was significantly higher in group O compared with group P and tended to be higher in group O compared with group C (Figure 3, *p* = 0.089). Moreover, the mRNA expression level of ABCG8 was significantly higher in group O than in groups C and P.

### Effect of exogenous PS or OPS on the mRNA expression of nuclear receptors involved in fatty acid and cholesterol metabolism in the liver

**FIGURE 4:**
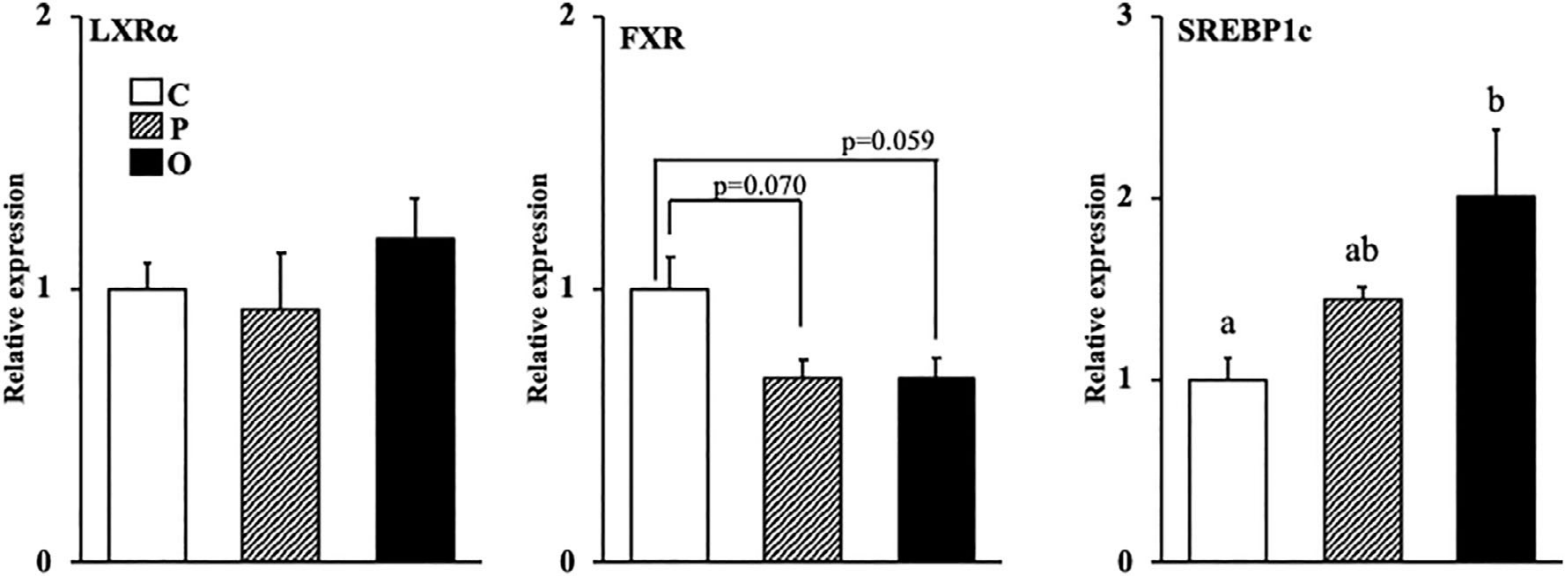
Effect of exogenous phytosterol or oxidized phytosterol on the mRNA expression of nuclear receptors involved in fatty acid and cholesterol metabolism in the liver. Data are presented as the mean ± SE of seven rats in each group. ^ab^Values without a common superscript letter are significantly different at *p* < 0.05. Abbreviations are the same as those in Table 1.

The mRNA expression level of SREBP1c was significantly higher in group O than in group C (Figure 4). The mRNA expression level of LXRα was not significantly different between the three groups. However, the mRNA expression level of FXR tended to be lower in groups P (*p* = 0.070) and O (*p* = 0.059) than in group C.

### Effect of PS or OPS on the micellar solubility of trilinolenin and cholesterol in vitro

**FIGURE 5:**
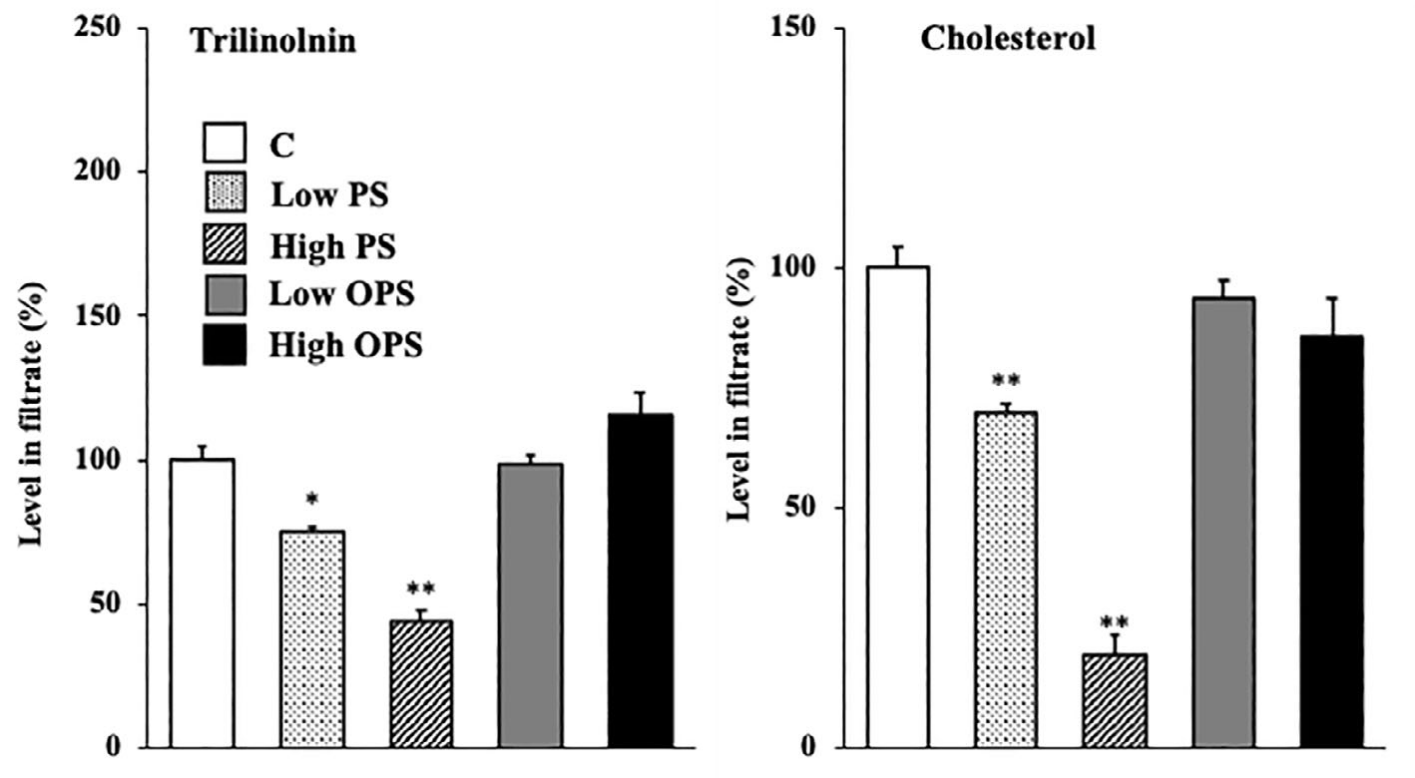
Effect of phytosterol or oxidized phytosterol on the micellar solubility of trilinolenin and cholesterol. Data are presented as the mean ± SE of four analyses in each group. Significant differences are compared with group C at *p < 0.05 and **p < 0.01. C, control; Low PS, micellar solution containing 1 mg PS; High PS, micellar solution containing 5 mg PS; Low OPS, micellar solution containing 1 mg OPS; high OPS, micellar solution containing 5 mg OPS.

The level of trilinolenin in the micellar solution was significantly lower in the presence of PS in a dose‐dependent manner but was unchanged in the presence of OPS (Figure 5). The level of cholesterol in the micellar solution was also significantly lower in the presence of PS in a dose‐dependent manner but was unchanged in the presence of OPS.

## DISCUSSION

Dietary PS exerts hypocholesterolemic action by inhibiting cholesterol absorption from the intestine, thereby reducing the risk of developing cardiovascular diseases (Katan et al., 2003). Thus, people are increasing their PS intake from daily diets by consuming PS‐enriched products or supplements containing high PS levels. PS is oxidized during heat processing or long‐term storage, resulting in the formation of various oxidized derivatives; hence, the intake level of OPS may increase in the future. The harmful effects of OPS in vivo, especially on fatty acid metabolism, are not fully understood. In the present study, we compared the effects of exogenous PS and OPS on essential fatty acid metabolism in rats.

We observed that exogenous OPS, but not PS, significantly inhibited body weight gain and the increase in white adipose tissue in rats, despite matching the same food intake under pair‐feeding conditions. Dietary OXC has been reported to inhibit the increase in body weight in rats through effects such as cytotoxicity and modulation of cholesterol metabolism (Osada et al., 1994, 1995). Therefore, OPS also may exhibit growth inhibition via its toxicity.

Sirle et al. reported that dietary PS lowered triacylglycerol levels in the liver by normalizing hepatic lipogenesis and β‐oxidation processes in dyslipidemic hamsters (Laos et al., 2014). Feng et al. also reported that dietary stigmasterol and β‐sitosterol decreased triacylglycerol levels in the liver of mice fed a high‐fat diet for 17 weeks (Feng et al., 2018). However, no significant change was observed in the liver triacylglycerol level in rats fed PS in the present study. This discrepancy could be attributed to species differences and dietary conditions. On the other hand, exogenous OPS significantly lowered the liver triacylglycerol levels. Previous studies have reported that dietary OXC lowers the liver triacylglycerol levels in rats (Osada et al., 1994, 1995). Ikeda et al. demonstrated that campestenone, an oxidized derivative of campesterol, activates the nuclear receptor peroxisome proliferator‐activated receptor α (PPARα; Ikeda et al., 2006). Therefore, OPS may exert agonistic effects on PPARα and then lower liver triacylglycerol level by promoting fatty acid β‐oxidation in the liver. Thus, absorbed OPS may also induce change in lipid parameters by activating PPARα because the absorption rate of OPS from intestine is higher than that of PS (Tomoyori et al., 2004). The effects of exogenous OPS on each nuclear receptor need to be examined in the future.

Exogenous PS significantly lowered total cholesterol levels in the liver, while exogenous OPS significantly lowered total cholesterol levels in both plasma and liver. Dietary PS is known to inhibit cholesterol absorption from the intestine by competitively inhibiting the solubilization of cholesterol into micelles, thereby lowering low‐density lipoprotein (LDL)‐C levels (Katan et al., 2003). However, Liang et al. did not observe this cholesterol‐lowering effect of dietary OPS in hamsters (Liang et al., 2011). Wang et al. also observed that while dietary PS lowered cholesterol levels in the plasma and liver of apoE‐deficient mice, OPS showed no such effect (Wang et al., 2021). The present study also observed that PS significantly lowered the micellar solubility of cholesterol, but OPS had no such effect. Thus, when oxidized, PS may lose its inhibitory effect on cholesterol absorption from the intestine. Liang et al. reported that dietary oxidized stigmasterol lowered the mRNA expression level of HMGCR in the liver of hamsters (Liang et al., 2011). 28‐Homobrassinolide, an OPS present in plants, has been found to lower the activity and gene expression of HMGCR by binding to the gene as a ligand (Mukherjee et al., 2016). Dietary OXC also causes the reduction of HMGCR activity in the liver and lowers cholesterol levels through its cytotoxicity (Osada et al., 1994, 1995). Previously, we demonstrated that dietary OPS significantly decreases the mRNA expression level of HMGCR and lowers the total cholesterol level in rat liver (Koyama et al., 2024). In the present study, exogenous OPS significantly lowered the mRNA expression of ABCG5 and ABCG8 in the liver. ABCG5 and ABCG8 are known as transporters of cholesterol excretion, and their mRNA expression is increased by the activation of LXR (Yu et al., 2003); PS is known to be an agonist of LXR (Bakrim et al., 2023). Exogenous OPS may act as a more potent agonist of LXR because the absorption rate of OPS from the intestine is higher than that of PS (Tomoyori et al., 2004) and may therefore lower total cholesterol levels by modulating transporters of cholesterol excretion by activating LXR. Thus, exogenous OPS may lower total cholesterol levels in the plasma and liver by modulating cholesterol metabolism through its ligand action or cytotoxic effect, rather than by inhibiting cholesterol absorption from the intestine.

Further, we found that exogenous OPS increased the mRNA expression and protein levels of both Δ6 and Δ5 desaturases. Linoleic acid is metabolized to γ‐linolenic acid by Δ6 desaturase and then converted to long‐chain unsaturated arachidonic acid by the action of ELOVL5 and Δ5 desaturase. Moreover, arachidonic acid is released from membrane phospholipids by PLA_2_ and oxidized by COX to produce eicosanoids such as leukotrienes and prostaglandins. We observed that exogenous OPS significantly increased the proportion of arachidonic acid among the total liver lipids, the Δ6 desaturation index in the total lipids of both liver and plasma, and the Δ5 desaturation index. Therefore, exogenous OPS may change the composition of essential fatty acids, especially n‐6 polyunsaturated fatty acids, by promoting the enzyme activity of Δ5 and Δ6 desaturase activities of Δ5 and Δ6 desaturase in the liver. We showed dietary OXC increased the level of arachidonic acid in liver phospholipids through modulation of linoleic acid metabolism by upregulating the hepatic Δ6 desaturase activity in rats (Osada et al., 1994, 1995). Moreover, we observed dietary OPS also increased the level of arachidonic acid in rat liver by modulating the mRNA expression of Δ6 desaturase (Koyama et al., 2024).

Previous in vitro studies have reported that the gene expressions of Δ5 desaturase, Δ6 desaturase, and ELOVL5 are increased by LXR and SREBP1c activity (Qin et al., 2009; Varin et al., 2015). Moreover, the gene expression of SREBP1c is modulated by LXR activity (Repa et al., 2000). Our study found no change in LXR gene expression in the liver, but the gene expression of SREBP1c was significantly higher in group O than in group C. As discussed previously, OPS may act as an agonist of LXR and modulate the expression of fatty acid desaturase through LXR activity. Moreover, it is well known that the target gene expression of LXR is modulated by the regulation of FXR activity (Watanabe et al., 2004). In the present study, the levels of αMCA and βMCA excreted in the feces were significantly higher in group O than in groups C and P. Moreover, OPS decreased gene expression of FXR in the liver. It has been reported that αMCA and βMCA (Chiang & Ferrell, 2022), as well as stigmasterol (Carter et al., 2007), act as antagonists of FXR. Additionally, OPS may also act as an antagonist of FXR either directly or indirectly through changes in bile acid composition in the intestine, or both. Thus, OPS may have enhanced SREBP1c gene expression by acting as an agonist of LXR or by inhibiting FXR activity, resulting in increased gene expression and protein levels of fatty acid desaturase. It is crucial to determine whether OPS acts as a ligand of LXR and FXR in the future. Triki et al. found that activation of complex 1 of the mammalian target of rapamycin (mTOR1) is involved in increasing the gene expression of Δ6 desaturase via the increase of SREBP activity (Triki et al., 2020). mTOR1 is known to be activated by oxidative stress (Yoshida et al., 2011). Oxidized β‐sitosterol exhibits cytotoxicity by causing oxidative stress on HepG2 cells (Koschutnig et al., 2009). Therefore, exogenous OPS may activate mTOR1 and then enhance Δ6 desaturase gene expression. This hypothesis should be verified in the future.

The increase of arachidonic acid level by exogenous OPS may promote atherosclerosis by promoting the production of prostaglandin, prostacyclin, and thromboxane related to endothelial dysfunction in the arteries. In fact, Yang et al. found that oxidized β‐sitosterol increased the production of prostanoids through upregulation of COX‐2 mRNA expression and attenuated the endothelium‐dependent vasorelaxation in rat aortic endothelial cells (Yang et al., 2013). Moreover, Plat et al. observed that dietary OPS increased the proportion of severe atherosclerotic lesions in LDL receptor‐deficient mice (Plat et al., 2014). Tomoyori et al. reported that dietary OPS increased the level of oxidized cholesterol in the serum of apoE‐deficient mice compared with PS‐fed mice (Tomoyori et al., 2004). In the present study, OPS had no effect on the mRNA expression levels of either PLA_2_ or COX‐2. However, OPS may increase the level of arachidonic acid, a substrate of eicosanoids, thereby increasing the level of eicosanoids derived from arachidonic acid. In general, changes in fatty acid composition of the liver are reflected in fatty acid composition of other tissues. Therefore, exogenous OPS may cause atherosclerosis in arteries through a variety of combined effects: imbalance of eicosanoid levels, promotion of cholesterol oxidation, and cytotoxicity.

On the other hand, Weingärtner et al. reported that dietary oxidized β‐sitosterol increased reactive oxygen species levels, but it did not increase atherosclerotic lesions in apoE‐deficient mice (Weingärtner et al., 2015). Tomoyori et al. also reported that dietary OPS did not promote atherosclerosis development in apoE‐deficient mice (Tomoyori et al., 2004). The reason for these contradictory results may be the differences in OPS composition, animal species, dietary conditions, and feeding periods in each study. Therefore, further studies on the change of eicosanoid levels in the plasma and artery and the development of atherosclerosis by exogenous OPS are required in the future.

Thus, we found that exogenous OPS modulates essential fatty acid metabolism. Some studies have reported that OPS exhibits cytotoxicity, induces inflammation, and causes pro‐atherogenic effects. Therefore, preventing the formation of OPS in processed foods and consuming proper PS‐rich health foods are essential.

## CONCLUSION

We compared the effects of exogenous PS and OPS on growth parameters, lipid metabolic parameters, and the desaturation of essential fatty acids in rats. Both exogenous OPS and PS lowered liver cholesterol levels; however, the reduction in plasma cholesterol levels was only observed in OPS‐fed rats. PS may lower liver cholesterol levels by inhibiting the micellar solubility of cholesterol. In contrast, OPS may lower liver cholesterol levels by upregulating the expression of the ABCG5/8 genes in the liver, as OPS lost its inhibitory action on the micellar solubility of cholesterol. Additionally, OPS increased the proportion of arachidonic acid by upregulating fatty acid desaturase, which was accompanied by the upregulation of SREBP1c gene expression in the liver. However, these effects were not observed in PS‐fed rats. Thus, exogenous PS and OPS have different effects on growth parameters and lipid metabolism.

## AUTHOR CONTRIBUTIONS

K.T. was involved in methodology, data collection, analysis, and manuscript writing. O.K. was involved in methodology, funding acquisition, supervision, and manuscript reviewing. All authors contributed to and approved the final draft of the manuscript.

## CONFLICT OF INTEREST STATEMENT

The authors declare that they have no conflict of interest.

## ETHICS STATEMENT

The animal experiments were conducted according to the guidelines of the Ethical Committee of Experimental Animal Care at Meiji University (approval code: MUIACUC2020‐01).

## Data Availability

The data that support the findings of this study are available from the corresponding author upon reasonable request.

## References

[lipd12444-bib-0001] Alemany L , Laparra JM , Barberá R , Alegría A . Relative expression of cholesterol transport‐related proteins and inflammation markers through the induction of 7‐ketosterol‐mediated stress in Caco‐2 cells. Food Chem Toxicol. 2013;56:247–253. 10.1016/j.fct.2013.02.040 23454145

[lipd12444-bib-0002] American Institute of Nutrition . Report of the American Institute of Nurtition ad hoc committee on standards for nutritional studies. J Nutr. 1977;107:1340–1348. 10.1093/jn/107.7.1340 874577

[lipd12444-bib-0003] Bakrim S , El Omari N , Khan EJ , Khalid A , Abdalla AN , Chook JB , et al. Phytosterols activating nuclear receptors are involving in steroid hormone‐dependent cancers: myth or fact? Biomed Pharmacother. 2023;169:115783. 10.1016/j.biopha.2023.115783 37944439

[lipd12444-bib-0004] Carter BA , Taylor OA , Prendergast DR , Zimmerman TL , Von Furstenberg R , Moore DD , et al. Stigmasterol, a soy lipid‐derived phytosterol, is an antagonist of the bile acid nuclear receptor FXR. Pediatr Res. 2007;62:301–306. 10.1203/pdr.0b013e3181256492 17622954

[lipd12444-bib-0005] Chiang JYL , Ferrell JM . Discovery of farnesoid X receptor and its role in bile acid metabolism. Mol Cell Endocrinol. 2022;548:111618. 10.1016/j.mce.2022.111618 35283218 PMC9038687

[lipd12444-bib-0006] Conchillo A , Cercaci L , Ansorena D , Rodriguez‐Estrada MT , Lercker G , Astiasarán I . Levels of phytosterol oxides in enriched and nonenriched spreads: application of a thin‐layer chromatography‐gas chromatography methodology. J Agric Food Chem. 2005;53:7844–7850. 10.1021/jf050539m 16190640

[lipd12444-bib-0007] Dutta PC . Studies on phytosterol oxides. II: content in some vegetable oils and in French fries prepared in these oils. J Am Oil Chem Soc. 1997;74:659–666. 10.1007/S11746-997-0198-6

[lipd12444-bib-0008] Dutta PC , Appeleqvist LA . Studies on phytosterol oxides I: effects of storage on the content in potato chips prepared in different vegetable oils. J Am Oil Chem Soc. 1997;74:647–657. 10.1007/s11746-997-0197-7

[lipd12444-bib-0009] Feng S , Dai Z , Liu AB , Huang J , Narsipur N , Guo G , et al. Intake of stigmasterol and β‐sitosterol alters lipid metabolism and alleviates NAFLD in mice fed a high‐fat western‐style diet. Biochim Biophys Acta Mol Cell Biol Lipids. 2018;1863:1274–1284. 10.1016/j.bbalip.2018.08.004 30305244 PMC6226309

[lipd12444-bib-0010] Folch J , Ascoli I , Lees M . Preparation of lipide extracts from brain tissue. J Biol Chem. 1951;191:833–841. 10.1016/S0021-9258(18)55987-1 14861228

[lipd12444-bib-0011] Gao J , Chen S , Zhang L , Cheng B , Xu A , Wu L , et al. Evaluation of cytotoxic and apoptotic effects of individual and mixed 7‐ketophytosterol oxides on human intestinal carcinoma cells. J Agric Food Chem. 2015;63:1035–1041. 10.1021/jf505079v 25542134

[lipd12444-bib-0012] Grandgirard A , Sergiel JP , Nour M , Demaison‐Meloche J , Giniès C . Lymphatic absorption of phytosterol oxides in rats. Lipids. 1999;34:563–570. 10.1007/s11745-999-0399-z 10405969

[lipd12444-bib-0013] Hirai K , Shimazu C , Takezoe R , Ozeki Y . Cholesterol, phytosterol and polyunsaturated fatty acid levels in 1982 and 1957 Japanese diets. J Nutr Sci Vitaminol. 1986;32:363–372. 10.3177/jnsv.32.363 3806251

[lipd12444-bib-0014] Ide T , Okamatsu H , Sugano M . Regulation by dietary fats of 3‐hydroxy‐3‐methylglutaryl‐coenzyme a reductase in rat liver. J Nutr. 1978;108:601–612. 10.1093/jn/108.4.601 632948

[lipd12444-bib-0015] Ikeda I , Konno R , Shimizu T , Ide T , Takahashi N , Kawada T , et al. Campest‐5‐en‐3‐one, an oxidized derivative of campesterol, activates PPARalpha, promotes energy consumption and reduces visceral fat deposition in rats. Biochim Biophys Acta. 2006;1760:800–807. 10.1016/j.bbagen.2006.02.017 16616424

[lipd12444-bib-0016] Katan MB , Grundy SM , Jones P , Law M , Miettinen T , Paoletti R , et al. Efficacy and safety of plant stanols and sterols in the management of blood cholesterol levels. Mayo Clin Proc. 2003;78:965–978. 10.4065/78.8.965 12911045

[lipd12444-bib-0017] Klingberg S , Andersson H , Mulligan A , Bhaniani A , Welch A , Bingham S , et al. Food sources of plant sterols in the EPIC Norfolk population. Eur J Clin Nutr. 2008;62:695–703. 10.1038/sj.ejcn.1602765 17440516

[lipd12444-bib-0018] Koschutnig K , Heikkinen S , Kemmo S , Lampi AM , Piironen V , Wagner KH . Cytotoxic and apoptotic effects of single and mixed oxides of beta‐sitosterol on HepG2‐cells. Toxicol In Vitro. 2009;23:755–762. 10.1016/j.tiv.2009.03.007 19328846

[lipd12444-bib-0019] Koyama T , Fukuoka D , Osada K . Effects of dietary oxidized phytosterol on lipid metabolism in rats. J Oleo Sci. 2024;73:1189–1199. 10.5650/jos.ess24064 39168626

[lipd12444-bib-0020] Lambelet P , Grandgirard A , Gregoire S , Juaneda P , Sebedio JL , Bertoli C . Formation of modified fatty acids and oxyphytosterols during refining of low erucic acid rapeseed oil. J Agric Food Chem. 2003;51:4284–4290. 10.1021/jf030091u 12848499

[lipd12444-bib-0021] Lampi AM , Juntunen L , Toivo J , Piironen V . Determination of thermo‐oxidation products of plant sterols. J Chromatogr B Analyt Technol Biomed Life Sci. 2002;777:83–92. 10.1016/S1570-0232(02)00094-6 12270202

[lipd12444-bib-0022] Laos S , Caimari A , Crescenti A , Lakkis J , Puiggròs F , Arola L , et al. Long‐term intake of soyabean phytosterols lowers serum TAG and NEFA concentrations, increases bile acid synthesis and protects against fatty liver development in dyslipidaemic hamsters. Br J Nutr. 2014;112:663–673. 10.1017/s0007114514001342 24932972

[lipd12444-bib-0023] Laparra JM , Alfonso‐García A , Alegría A , Barberá R , Cilla A . 7keto‐stigmasterol and 7keto‐cholesterol induce differential proteome changes to intestinal epitelial (Caco‐2) cells. Food Chem Toxicol. 2015;84:29–36. 10.1016/j.fct.2015.06.021 26140950

[lipd12444-bib-0024] Leal‐Castañeda EJ , Inchingolo R , Cardenia V , Hernandez‐Becerra JA , Romani S , Rodriguez‐Estrada MT , et al. Effect of microwave heating on phytosterol oxidation. J Agric Food Chem. 2015;63:5539–5547. 10.1021/acs.jafc.5b00961 25973984

[lipd12444-bib-0025] Liang YT , Wong WT , Guan L , Tian XY , Ma KY , Huang Y , et al. Effect of phytosterols and their oxidation products on lipoprotein profiles and vascular function in hamster fed a high cholesterol diet. Atherosclerosis. 2011;219:124–133. 10.1016/j.atherosclerosis.2011.06.004 21719014

[lipd12444-bib-0026] Luu W , Sharpe LJ , Capell‐Hattam I , Gelissen IC , Brown AJ . Oxysterols: old tale, new twists. Annu Rev Pharmacol Toxicol. 2016;56:447–467. 10.1146/annurev-pharmtox-010715-103233 26738477

[lipd12444-bib-0027] Menéndez‐Carreño M , Ansorena D , Astiasarán I . Stability of sterols in phytosterol‐enriched milk under different heating conditions. J Agric Food Chem. 2008;56:9997–10002. 10.1021/jf802000m 18928298

[lipd12444-bib-0028] Menéndez‐Carreño M , García‐Herreros C , Astiasarán I , Ansorena D . Validation of a gas chromatography‐mass spectrometry method for the analysis of sterol oxidation products in serum. J Chromatogr B Analyt Technol Biomed Life Sci. 2008;864:61–68. 10.1016/j.jchromb.2008.01.036 18272439

[lipd12444-bib-0029] Morin RJ , Hu B , Peng SK , Sevanian A . Cholesterol oxides and carcinogenesis. J Clin Lab Anal. 1991;5:219–225. 10.1002/jcla.1860050312 2061746

[lipd12444-bib-0030] Mukherjee V , Vijayalaksmi D , Gulipalli J , Premalatha R , Sufi SA , Velan A , et al. A plant oxysterol, 28‐homobrassinolide binds HMGCoA reductase catalytic cleft: stereoselective avidity affects enzyme function. Mol Biol Rep. 2016;43:1049–1058. 10.1007/s11033-016-4052-5 27585573

[lipd12444-bib-0031] Oehrl LL , Hansen AP , Rohrer CA , Fenner GP , Boyd LC . Oxidation of phytosterols in a test food system. J Am Oil Chem Soc. 2001;78:1073–1078. 10.1007/s11746-001-0391-z

[lipd12444-bib-0032] Ogino Y , Osada K , Nakamura S , Ohta Y , Kanda T , Sugano M . Absorption of dietary cholesterol oxidation products and their downstream metabolic effects are reduced by dietary apple polyphenols. Lipids. 2007;42:151–161. 10.1007/s11745-006-3008-2 17393221

[lipd12444-bib-0033] Osada K , Kodama T , Cui L , Ito Y , Sugano M . Effects of dietary oxidized cholesterol on lipid metabolism in differently aged rats. Biosci Biotechnol Biochem. 1994;58:1062–1069. 10.1271/bbb.58.1062

[lipd12444-bib-0034] Osada K , Kodama T , Noda S , Yamada K , Sugano M . Oxidized cholesterol modulates age‐related change in lipid metabolism in rats. Lipids. 1995;30:405–413. 10.1007/bf02536298 7637560

[lipd12444-bib-0035] Ostlund RE Jr . Phytosterols and cholesterol metabolism. Curr Opin Lipidol. 2004;15:37–41. 10.1097/00041433-200402000-00008 15166807

[lipd12444-bib-0036] Peng SK , Taylor CB , Hill JC , Morin RJ . Cholesterol oxidation derivatives and arterial endothelial damage. Atherosclerosis. 1985;54:121–133. 10.1016/0021-9150(85)90172-8 3986012

[lipd12444-bib-0037] Plat J , Theuwissen E , Husche C , Lütjohann D , Gijbels MJ , Jeurissen M , et al. Oxidised plant sterols as well as oxycholesterol increase the proportion of severe atherosclerotic lesions in female LDL receptor+/− mice. Br J Nutr. 2014;111:64–70. 10.1017/s0007114513002018 23773414

[lipd12444-bib-0038] Qin Y , Dalen KT , Gustafsson JA , Nebb HI . Regulation of hepatic fatty acid elongase 5 by LXRalpha‐SREBP‐1c. Biochim Biophys Acta. 2009;1791:140–147. 10.1016/j.bbalip.2008.12.003 19136075

[lipd12444-bib-0039] Repa JJ , Liang G , Ou J , Bashmakov Y , Lobaccaro JM , Shimomura I , et al. Regulation of mouse sterol regulatory element‐binding protein‐1c gene (SREBP‐1c) by oxysterol receptors, LXRalpha and LXRbeta. Genes Dev. 2000;14:2819–2830. 10.1101/gad.844900 11090130 PMC317055

[lipd12444-bib-0040] Ryan E , Chopra J , McCarthy F , Maguire AR , O'Brien NM . Qualitative and quantitative comparison of the cytotoxic and apoptotic potential of phytosterol oxidation products with their corresponding cholesterol oxidation products. Br J Nutr. 2005;94:443–451. 10.1079/BJN20051500 16176617

[lipd12444-bib-0041] Scholz B , Guth S , Engel KH , Steinberg P . Phytosterol oxidation products in enriched foods: occurrence, exposure, and biological effects. Mol Nutr Food Res. 2015;59:1339–1352. 10.1002/mnfr.201400922 25787244

[lipd12444-bib-0042] Sevanian A , Peterson AR . The cytotoxic and mutagenic properties of cholesterol oxidation products. Food Chem Toxicol. 1986;24:1103–1110. 10.1016/0278-6915(86)90295-4 3804113

[lipd12444-bib-0043] Sugano M , Yamada Y , Yoshida K , Hashimoto Y , Matsuo T , Kimoto M . The hypocholesterolemic action of the undigested fraction of soybean protein in rats. Atherosclerosis. 1988;72:115–122. 10.1016/0021-9150(88)90071-8 3063266

[lipd12444-bib-0044] Tomoyori H , Kawata Y , Higuchi T , Ichi I , Sato H , Sato M , et al. Phytosterol oxidation products are absorbed in the intestinal lymphatics in rats but do not accelerate atherosclerosis in apolipoprotein E‐deficient mice. J Nutr. 2004;134:1690–1696. 10.1093/jn/134.7.1690 15226455

[lipd12444-bib-0045] Triki M , Rinaldi G , Planque M , Broekaert D , Winkelkotte AM , Maier CR , et al. mTOR signaling and SREBP activity increase FADS2 expression and can activate sapienate biosynthesis. Cell Rep. 2020;31:107806. 10.1016/j.celrep.2020.107806 32579932 PMC7326293

[lipd12444-bib-0046] Varin A , Thomas C , Ishibashi M , Ménégaut L , Gautier T , Trousson A , et al. Liver X receptor activation promotes polyunsaturated fatty acid synthesis in macrophages: relevance in the context of atherosclerosis. Arterioscler Thromb Vasc Biol. 2015;35:1357–1365. 10.1161/atvbaha.115.305539 25838428

[lipd12444-bib-0047] Wang M , Yang B , Shao P , Jie F , Yang X , Lu B . Sterols and sterol oxidation products: effect of dietary intake on tissue distribution in ApoE‐deficient mice. J Agric Food Chem. 2021;69:11867–11877. 10.1021/acs.jafc.1c03648 34586790

[lipd12444-bib-0048] Watanabe M , Houten SM , Wang L , Moschetta A , Mangelsdorf DJ , Heyman RA , et al. Bile acids lower triglyceride levels via a pathway involving FXR, SHP, and SREBP‐1c. J Clin Invest. 2004;113(10):1408–1418. 10.1172/jci21025 15146238 PMC406532

[lipd12444-bib-0049] Weingärtner O , Husche C , Schött HF , Speer T , Böhm M , Miller CM , et al. Vascular effects of oxysterols and oxyphytosterols in apoE −/− mice. Atherosclerosis. 2015;240:73–79. 10.1016/j.atherosclerosis.2015.02.032 25765595

[lipd12444-bib-0050] Yang C , Chen ZY , Wong SL , Liu J , Liang YT , Lau CW , et al. Β‐sitosterol oxidation products attenuate vasorelaxation by increasing reactive oxygen species and cyclooxygenase‐2. Cardiovasc Res. 2013;97:520–532. 10.1093/cvr/cvs370 23250922

[lipd12444-bib-0051] Yoshida S , Hong S , Suzuki T , Nada S , Mannan AM , Wang J , et al. Redox regulates mammalian target of rapamycin complex 1 (mTORC1) activity by modulating the TSC1/TSC2‐Rheb GTPase pathway. J Biol Chem. 2011;286:32651–32660. 10.1074/jbc.m111.238014 21784859 PMC3173157

[lipd12444-bib-0052] Yu L , York J , von Bergmann K , Lutjohann D , Cohen JC , Hobbs HH . Stimulation of cholesterol excretion by the liver X receptor agonist requires ATP‐binding cassette transporters G5 and G8. J Biol Chem. 2003;278(18):15565–15570. 10.1074/jbc.m301311200 12601003

